# Cinnamaldehyde nanoemulsion-loaded pectin films for delaying quality deterioration of fresh-cut chili pepper during refrigerated storage: Influence of pectin esterification and nanoemulsion concentration

**DOI:** 10.1016/j.fochx.2026.104469

**Published:** 2026-09-14

**Authors:** Zudi Li, Xiaoyan Zhao, Qingyu Yang, Dan Wang, Pan Wang, Shuang Zhao, Wenting Zhao

**Affiliations:** a Institute of Agri-food Processing and Nutrition, Beijing Academy of Agriculture and Forestry Sciences, Beijing Key Laboratory of Agricultural Products of Fruits and Vegetables Preservation and Processing, Key Laboratory of Vegetable Postharvest processing, Ministry of Agriculture and Rural Affairs, Beijing 100097, China; b College of Grain Science and Technology, Shenyang Normal University, Shenyang 110034, China

**Keywords:** Nanoemulsion, Active packaging, Pectin, Antibacterial activity, *Trans*-cinnamaldehyde, Fresh-cut chili pepper

## Abstract

The objective of this study was to fabricate antibacterial pectin-based active films activated with *trans*-cinnamaldehyde delivered through a nanoemulsion system. A whey protein isolate-inulin complex-stabilized nanoemulsion was incorporated into high-methoxyl pectin (HMP) and low-methoxyl pectin (LMP) matrices at four levels (0, 5, 17.5, and 30% *w*/w). The film morphology, physicochemical properties, *trans*-cinnamaldehyde retention, and antibacterial activity against *Enterobacter cloacae* were systematically investigated. Average droplet diameter and ζ-potential of the nanoemulsion were 162.77 ± 1.07 nm and − 30.08 ± 0.48 mV, respectively. Hydrogen bonding, electrostatic forces, and hydrophobic associations synergistically facilitated the assembly of a compact film network. Increasing emulsion content significantly enhanced surface hydrophobicity, water vapor and ultraviolet shielding capacity, and antibacterial efficacy. Films based on HMP exhibited superior UV-blocking performance, enhanced hydrophobicity, more controlled release of *trans*-cinnamaldehyde, and stronger antimicrobial activity. Furthermore, HMP-based active films effectively maintained visual quality and delayed firmness loss in fresh-cut chili peppers throughout refrigerated storage. Collectively, the fabricated *trans*-cinnamaldehyde-loaded pectin films demonstrated great potential as antibacterial active packaging for perishable vegetables.

## Highlights

• Pectin films loaded with trans-cinnamaldehyde nanoemulsion were developed.
• Emulsion loading improved hydrophobicity, UV and water vapor barriers.
• HMP films showed better sustained release and antimicrobial performance.
• HMP film with 30% emulsion best preserved fresh-cut chili peppers.
• The active films offer promise for extending fresh-cut produce shelf life.

## Graphical abstract

**Figure:**
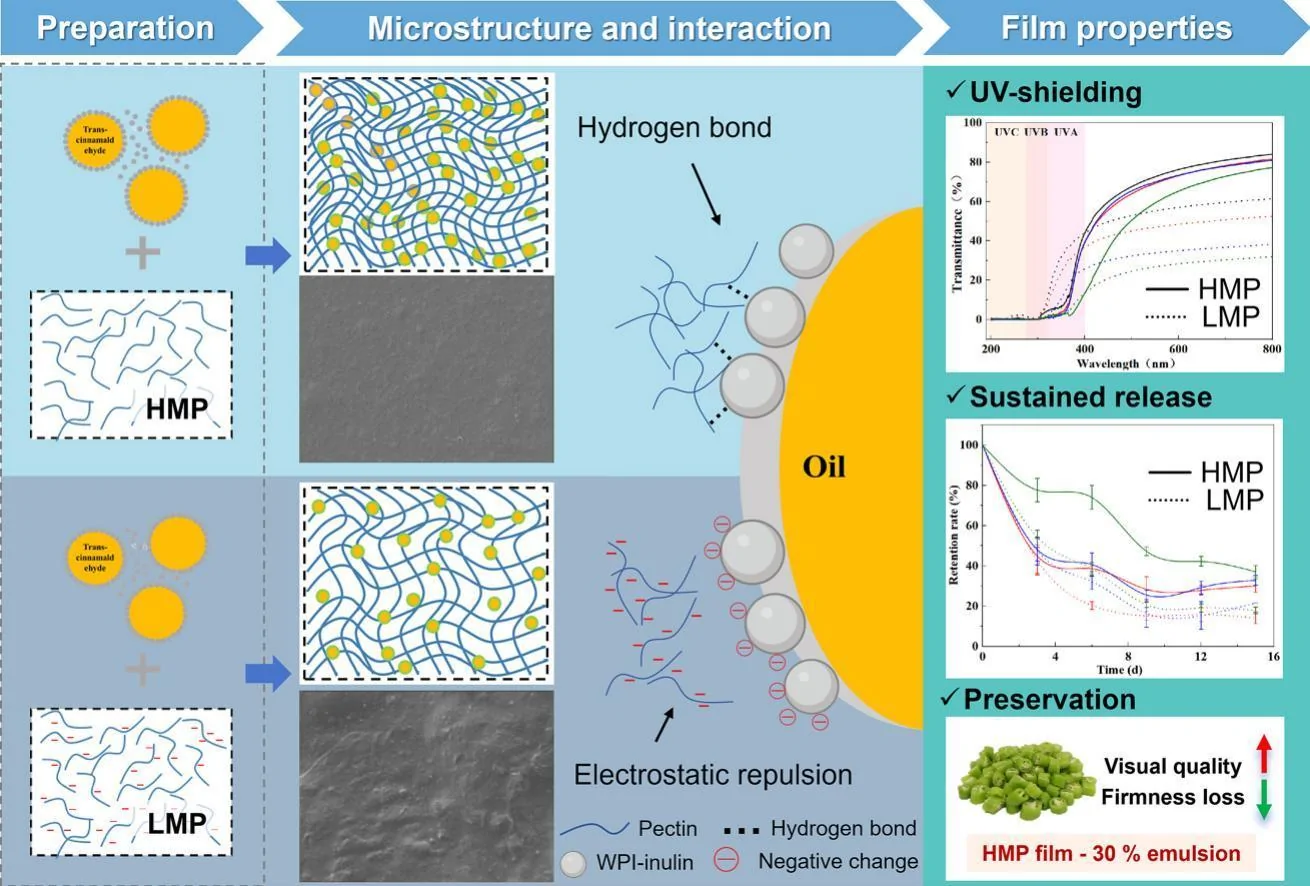

## 1 Introduction

Traditional petroleum-based plastic remains prevalent in packaging for food products, but its environmental impact is substantial due to limited biodegradability, poor recyclability, and persistence in ecosystems as microplastic residues (Zhang et al., 2023). In response, there has been growing interest in sustainable alternatives composed of renewable biopolymers, particularly proteins and polysaccharides, which offer inherent biodegradability, reduced carbon footprints, and compatibility with circular economy principles (Zhao et al., 2025). Among emerging strategies, active biopolymer-based packaging has gained prominence by enabling the sustained release of functional compounds from packaging matrix into food microenvironment, hence prolonging shelf life without direct food additives (Liu et al., 2024). Consequently, the integration of bioactive functionalities into biodegradable hydrocolloid films represents a synergistic approach to concurrently enhance food preservation efficacy and mitigate plastic pollution, a dual objective aligned with current sustainability imperatives in food packaging innovation.

*trans*-Cinnamaldehyde, a major volatile compound of cinnamon essential oil, is commonly used in food preservation to provide green, food-grade antimicrobial and antibiofilm protection (Mondéjar-López et al., 2024). It has demonstrated potent inhibitory activity against a broad spectrum of both Gram-positive and Gram-negative bacteria (Baghi et al., 2024). However, its direct application in fresh produce systems is limited by its high volatility and susceptibility to degradation under light and heat, leading to rapid loss of bioactivity during processing and storage (Baghi et al., 2023). Additionally, its strong aroma and chemical reactivity may adversely affect sensory quality of the produce (Li et al., 2023).

Nanoemulsion-based delivery systems offer an effective strategy to overcome these limitations by encapsulating essential oil, enabling controlled release, prolonged activity, and protection from environmental degradation (Bolgen et al., 2025). Among biopolymer-based emulsifiers, protein-polysaccharide complexes have garnered significant attention due to their synergistic interfacial activity, improved steric/electrostatic stabilization, and capacity to modulate release profiles. In particular, whey protein isolate (WPI)-inulin complex has been demonstrated to serve as an excellent carrier that synergistically enhances both the stability of bioactive compounds and the colloidal integrity of emulsion systems (Li, Zhou, et al., 2024). WPI is a globular, amphiphilic, dairy-derived protein with excellent emulsifying and gelling properties (Deng et al., 2024). Inulin, a linear *β-*(2 → 1)-linked fructan, acts not merely as a bulking agent but as a co-stabilizer in such system, adsorbing onto protein-coated droplet interfaces to form a denser, more resilient barrier via non-covalent interactions (e.g., hydrogen bonding, excluded volume effects). In addition, pectin, a naturally abundant and anionic plant polysaccharide composed primarily of D-galacturonic acid residues, has emerged as a compelling matrix for biodegradable active films due to its superior film-forming capacity, biocompatibility, and responsiveness to structural modification (Yang et al., 2024). Importantly, pectin's functional properties are governed by its degree of esterification (DE), which dictates chain conformation, charge density, and gelation mechanism. High-methoxyl pectin (HMP) with DE > 50% undergoes acid- and sugar-mediated hydrophobic association, whereas low-methoxyl pectin (LMP) with DE < 50% relies on calcium-mediated ionic crosslinking (“egg-box” model) (Muhoza et al., 2019). These distinct network architectures profoundly influence mechanical strength, water barrier properties, and diffusion kinetics of embedded actives, enabling tailored design of delivery films (Naqash et al., 2017). Nevertheless, despite advances in pectin-based packaging and nanoemulsion stabilization, the systematic exploration of *trans*-cinnamaldehyde-loaded WPI-inulin nanoemulsion embedded in pectin films with different DE has not been conducted. Crucially, the interplay between pectin DE, nanoemulsion-matrix compatibility, and the resultant release dynamics, antimicrobial performance, and preservation efficacy, particularly for highly perishable produce such as fresh-cut fruit and vegetables, remains poorly understood.

Fresh-cut chili peppers (*Capsicum annuum* L) have gained considerable attention in the ready-to-eat market, owing to its pungent flavor profile, high antioxidant and bioactive content, and consumer demand for convenient, minimally processed produce (Li et al., 2024a; Li et al., 2025a). Nevertheless, mechanical wounding during cutting operation compromises tissue integrity, leading to elevated respiration rates, increased ethylene biosynthesis, and membrane lipid peroxidation, collectively accelerating textural softening and nutrient loss (Song et al., 2025). Importantly, the exposed tissues create nutrient-rich entry points for microorganisms, with *Enterobacter cloacae* (*E. cloacae*) recently identified as a dominant spoilage bacteria in stored fresh-cut chili peppers that directly correlated with juice exudation and loss of firmness (Li et al., 2024a, 2025b). Given the limited efficacy of conventional cold-chain management alone in controlling such spoilage microbiota, the development of targeted and efficient preservation strategies is urgently needed to prolong shelf life and keep freshness of fresh-cut peppers.

In this research, *trans*-cinnamaldehyde-loaded nanoemulsions, stabilized by WPI-inulin complexes, were incorporated into HMP and LMP films at varying loadings. The resulting active films were comprehensively characterized to elucidate the interplay between nanoemulsion content and pectin degree of esterification on key functional attributes, including physicochemical performance, morphological properties, gas permeability, release kinetics of *trans*-cinnamaldehyde, as well as antibacterial efficacy against *E. cloacae*. Subsequently, the preservation performance of selected films was evaluated on fresh-cut chili peppers over refrigerated storage. This work provides a sustainable and effective strategy to delay quality deterioration of fresh-cut produce, offering new insights into structure-function correlations of pectin-based active packaging.

## 2 Material and methods

### 2.1 Materials

WPI, inulin, and *trans*-cinnamaldehyde were provided by Yuanye Bio-Tech Co., Ltd. (Shanghai, China). Glycerol was supplied by Biotopped Technology Co., Ltd. (Beijing, China). Citrus pectin (HMP, DE = 65.32%) and sunflower pectin (LMP, DE = 33.27%) were provided by Sigma-Aldrich (Germany) and Constan Biotech Co., Ltd. (Inner Mongolia, China), respectively.

### 2.2 Preparation of nanoemulsion

WPI (4%) and inulin (4%) were separately dissolved in deionized water (Almasi et al., 2020). The two solutions were subsequently blended (1:1, *w*/w) and allowed to hydrate for 24 h to ensure complete hydration. Subsequently, *trans*-cinnamaldehyde was then gradually introduced into the biopolymer mixture under continuous stirring to form a coarse pre-emulsion, corresponding to a ratio of 1:4 (*trans*-cinnamaldehyde: WPI-inulin mixture). The pre-emulsion was further homogenized for 4 min at 20,500 rpm to obtain the stable nanoemulsion.

### 2.3 Average droplet size and ζ-potential

ζ-potential and average droplet size of the nanoemulsion were characterized via dynamic light scattering (Zetasizer Nano ZS90, Malvern, UK) (Su et al., 2026).

### 2.4 Preparation of active films

Pectin-based active film was produced using a previously reported approach, as described by Almasi et al. (2020). Pectin solution was formulated by dissolving HMP or LMP in deionized water (3%), followed by agitating under ambient conditions for 18 h until complete dissolution was achieved. Subsequently, the *trans*-cinnamaldehyde-loaded emulsion was incorporated to pectin solutions with varying mass ratios (0, 5, 17.5, 30%) with magnetic stirring for 15 min. Then glycerol was introduced with a ratio of 40% (*w*/w, based on pectin content) and mixed thoroughly. To eliminate residual air bubbles, the film-forming solutions were degassed under reduced pressure using a VC-50 vacuum system (Renggli AG., Rotkreuz, Switzerland) for 30 min. Film formation was achieved by pouring the solutions (60 g) onto glass Petri dishes (15 cm diameter), then drying at 25 °C over 48 h. The casting mass of 60 g was selected based on a previously reported method (Almasi et al., 2020) and preliminary trials to achieve a uniform film avoiding film brittleness or over-flexibility. After drying, the film was peeled-off and equilibrated at 55% relative humidity and 25 °C for 72 h prior to characterization. The resulting films were designated as HMP0, HMP5, HMP17.5, HMP30, LMP0, LMP5, LMP17.5, and LMP30, corresponding to the pectin type and emulsion concentration.

### 2.5 Characterization of composite films

#### 2.5.1 Fourier transform infrared spectroscopy (FTIR)

FTIR spectra were collected using a Nicolet iS20 spectrometer (Thermo Fisher Scientific, USA). Spectra were recorded within the wavenumber range of 4000–400 cm^−1^, with 32 accumulated scans at a spectral resolution of 4 cm^−1^. Samples were prepared as KBr pellets for FTIR analysis.

#### 2.5.2 X-ray diffraction (XRD)

Crystalline characteristics were recorded using X-ray diffractometer (SmartLab SE, Rigaku, Japan) in the diffraction angle (2θ) range of 5°-50° (Nisar et al., 2018).

#### 2.5.3 Scanning electron microscopy (SEM)

Microstructure was recorded using a scanning electron microscope (MIRA LMS, TESCAN, Shanghai, China). For morphological characterization of the cross-sectional microstructure, film samples were fractured in liquid nitrogen to generate clean cross-section.

#### 2.5.4 Optical properties

For optical characterization, the film was sectioned into rectangular samples (40 mm × 10 mm), put into a UV cell, and then scanned using a UV–Vis spectrophotometer (UV-1800, Shimadzu, Japan), with air used as the reference (Zhang et al., 2023). The transmittance was evaluated in the wavelength range of 200–800 nm.

#### 2.5.5 Film thickness

The thickness of film samples was determined using a thickness gauge (CHY-C2, Labthink, China) at five different positions on each film, and the average value was calculated.

#### 2.5.6 Gas permeability measurement

The films were cut into circular pieces using a special cylindrical sample. Water vapor permeability (WVP) was quantified at 38 °C and 90% relative humidity using a WVP analyzer (W3–031, Labthink, China), with samples equilibrated for 1 h prior to testing and mass changes recorded every 0.5 h. The O_2_ and CO_2_ were assessed using a VAC-V2 gas permeability analyzer (Labthink, China).

#### 2.5.7 Water contact angle measurement

Water contact angle (WCA) was evaluated using a surface tension tester (JY-82C, Chengdedingsheng, China). Film was positioned on the test platform, following which a droplet of distilled water was applied to the film surface by a microsyringe. Test photographs were taken and WCA was calculated according to Liu et al. (2021).

#### 2.5.8 Release properties

According to the methods of (Zhang et al., 2022), the release of *trans*-cinnamaldehyde from films was analyzed via headspace solid-phase microextraction (SPME) combined with GC–MS (Agilent 5977B, USA) and a 30 m × 0.25 mm × 0.25 μm MS column (HP-5, Agilent, USA). Briefly, 3.0 mg of film was sealed in a 20 mL vial and incubated at 80 °C for 30 min. Volatiles were adsorbed onto an SPME fiber at 80 °C for 5 min, then desorbed at 250 °C for 5 min (split ratio 100:1). The GC oven program: 50 °C (1 min), ramped to 250 °C at 8 °C·min^−1^, then to 300 °C at 35 °C·min^−1^ (held 5 min); helium carrier gas flowed at 1.2 mL·min^−1^. MS conditions: EI mode (70 eV), ion source at 230 °C, *m*/*z* 33–500. *trans*-Cinnamaldehyde retention was calculated as the ratio of residual to initial peak area. Films were stored at room temperature, and analyses were performed on days 0, 3, 6, 9, and 12.

#### 2.5.9 Antibacterial performance analysis

The antimicrobial performance was determined using *E. cloacae* as the indicator microorganism, following Liu et al. (2021) with minor adjustments. Film (100 mg) was immersed into 5 mL of BHI broth containing 20 μL of an *E. cloacae* inoculum (approximately 10^8^ CFU·mL^−1^). Incubation was carried out at 37 °C under shaking conditions (210 rpm) for 10 h. To analyze microbial growth at different culture times, the cultured mixed system was serially diluted with sterile saline and plated on BHI agar for colony enumeration. At the same time, an equal amount of bacterial suspension and sterile medium were employed as the positive and negative controls, respectively.

### 2.6 Preservation performance of fresh-cut chili peppers

Fresh-cut chili peppers (Hangjiao No.2, *Capsicum annuum* L.) were prepared following the method described in our previous work (Li et al., 2025a). The fresh-cut chili peppers (80 g) from the same batch were placed in polypropylene trays (300 mL), and then sealed with either pectin-based films or commercial ethylene-vinyl alcohol copolymer (EVOH) wrap (Xuri Packaging Co., Ltd., China) of dimensions 10.8 cm by 8.3 cm. The unpackaged samples were served as the control group. All groups were refrigerated for 10 days at 10 °C. Visual quality was recorded and firmness was measured using a texture analyzer based on Li et al. (2024b).

### 2.7 Statistical analysis

All experiments were repeated in triplicate. Results were presented as mean ± standard deviation. Data were subjected to one-way ANOVA in SPSS software (version 17.0), followed by Duncan's multiple range test to identify significant differences (*p* < 0.05).

## 3 Results and discussion

### 3.1 Nanoemulsion characterization

The droplet size and ζ-potential of the nanoemulsion are showed in Fig. S1. Droplet size plays an essential role in determining emulsion stability, encapsulated compound release behavior, and the functional performance of emulsion-based films (Almasi et al., 2020). Average droplet diameter of the *trans*-cinnamaldehyde-loaded nanoemulsion was 162.77 ± 1.07 nm, which was larger than the 97.5 nm reported for marjoram essential oil-loaded nanoemulsions (Almasi et al., 2020). Such discrepancies are likely attributable to variations in hydrophobicity of essential oil, as well as differences in homogenization intensity and formulation design.

The polydispersity index (PDI) of the nanoemulsion was 0.05, indicating a narrow size distribution and high droplet uniformity. PDI values below 0.4 are generally associated with well-dispersed and stable nanoemulsion systems suitable for incorporation into film matrices (Acharya et al., 2025). The ζ-potential of nanoemulsion was −30.08 ± 0.48 mV, which was comparable to values reported for WPI-inulin stabilized emulsions (approximately −34 mV) (Bakry et al., 2017). This relatively high negative charge suggests sufficient electrostatic repulsion among droplets, thereby enhancing resistance to aggregation and flocculation phenomena (Acevedo-Fani et al., 2015; Pérez-Córdoba et al., 2018).

### 3.2 Characterization of active pectin films

#### 3.2.1 FTIR spectroscopy

**Fig. 1:**
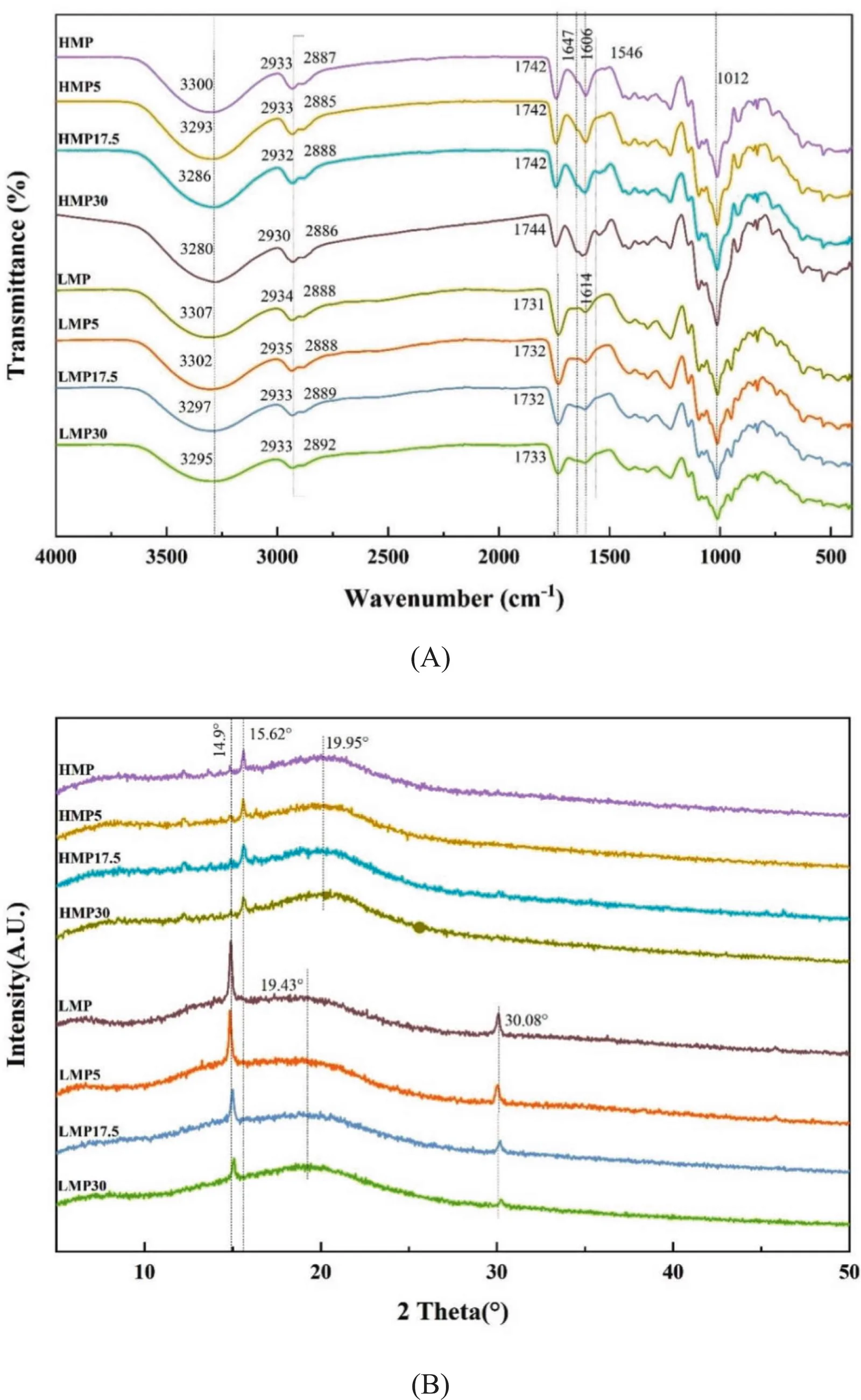
FT-IR spectra (A) and XRD patterns (B) of pectin-based films activated by *trans*-cinnamaldehyde-loaded nanoemulsion.

Fourier-transform infrared (FTIR) spectroscopy was employed to elucidate molecular interactions in the pectin-based film matrix. As shown in Fig. 1A, both HMP and LMP alone films displayed a broad absorption band centered near 3300 cm^−1^, characteristic of O—H stretching vibrations from hydroxyl groups in pectin. Characteristic bands at 2887 cm^−1^ and 2933 cm^−1^ were observed, corresponding to the C—H stretching vibrations (Lingait & Kumar, 2025). Bands in the range of 1731–1742 cm^−1^ were assigned to esterified carboxyl (-COOCH_3_) and protonated carboxylic acid (-COOH) stretching vibrations, while bands at 1606–1614 cm^−1^ represented asymmetric stretching of deprotonated carboxylate groups (-COO^−^) (Sood & Saini, 2022; Zhang et al., 2022). Furthermore, a characteristic absorption band at approximately 1012 cm^−1^ was assigned to C-O-C stretching vibrations associated with glycosidic linkages in the pectin backbone.

In comparison to pectin films without emulsion, the broad O—H stretching band showed a progressive change toward lower wavenumbers upon incorporation of *trans*-cinnamaldehyde-loaded nanoemulsion. This redshift intensified with increasing nanoemulsion concentration, revealing progressively stronger hydrogen bonding between pectin hydroxyl groups and polar functional moieties (e.g., -OH, C=O) present in the WPI-inulin complex and/or cinnamaldehyde molecules (Bhatia et al., 2024; Sood et al., 2022). In addition, two new absorption bands emerged around 1546 cm^−1^ and 1647 cm^−1^ in emulsion-containing films, corresponding to the N—H bending (amide II) and C=O stretching (amide I) of WPI, respectively (Liu et al., 2020), confirming the effective integration of WPI into the pectin-based film network. Concurrently, the peak intensity of deprotonated COO^−^ groups at 1606–1614 cm^−1^ increased, suggesting enhanced electrostatic interactions between whey protein isolate and pectin (Zhang et al., 2022). Furthermore, the absorption band at 1012 cm^−1^ showed a progressive decrease in intensity with increasing emulsion content. This attenuation is likely attributable to the combined effects of enhanced hydrogen bonding between pectin hydroxyl/carboxyl groups and polar residues of WPI, as well as strengthened hydrophobic interactions involving nonpolar amino acids like valine, leucine, and isoleucine in WPI (Zhang et al., 2022). Therefore, the changes in FTIR spectra caused by adding emulsion to pectin were due to hydrogen bonds and electrostatic interactions in the samples, as well as the hydrophobic interactions induced by hydrophobic amino acids.

Notably, compared to LMP-based films, HMP films exhibited distinct spectral responses to increasing nanoemulsion concentration. While the intensity of the carboxylate (COO^−^) vibrations at 1606–1614 cm^−1^ remained essentially unchanged in HMP films, a marked decrease was observed for LMP films. Additionally, HMP films showed a greater redshift in the O—H stretching band (∼3300 cm^−1^) and a stronger attenuation of the C-O-C glycosidic linkage band (∼1012 cm^−1^). These differences suggest enhanced intermolecular interactions and superior compatibility between the nanoemulsion and the HMP matrix (Guo et al., 2020).

#### 3.2.2 XRD analysis

Crystallinity of the films was analyzed by XRD, which is closely related to their physicochemical characteristics. Distinct diffraction peaks in the XRD patterns are indicative of crystalline domains, whereas their absence reflects an amorphous structure (Bello & Peresin, 2024). Fig. 1B showed pure pectin and emulsion-containing films displayed similar diffraction patterns, differing mainly in peak intensity and sharpness. HMP and LMP alone films exhibited broad diffraction peaks centered around 2θ = 19.95° and 19.43°, respectively. Incorporation of the nanoemulsion did not shift the position of these principal peaks but slightly led to peak narrowing and increased intensity, particularly in HMP-based films. This suggests that the nanoemulsion was well incorporated within the pectin matrix without disrupting its semi-crystalline structure (Zhang et al., 2022). The enhanced diffraction intensity reflects strengthened intermolecular interactions including electrostatic associations and hydrogen bonding, consistent with FTIR observations (Wang et al., 2020). Additional crystalline peaks were observed at 2θ = 15.62° for HMP and at 2θ = 14.9° and 30.08° for LMP. Upon emulsion incorporation, attenuation of these secondary peaks was observed, reflecting successful complex formation between pectin and the WPI-stabilized nanoemulsion (Su et al., 2026).

#### 3.2.3 SEM

**Fig. 2:**
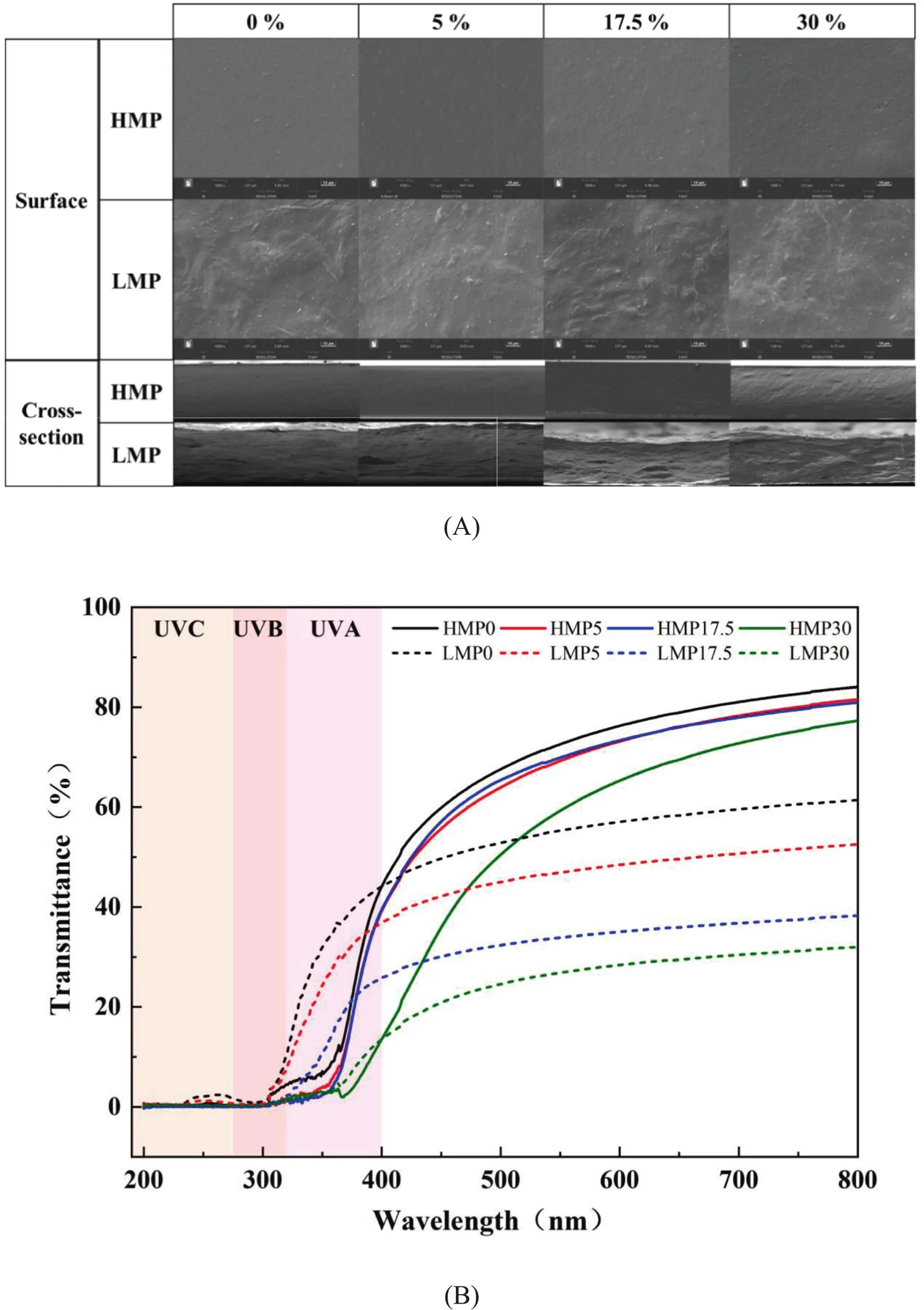

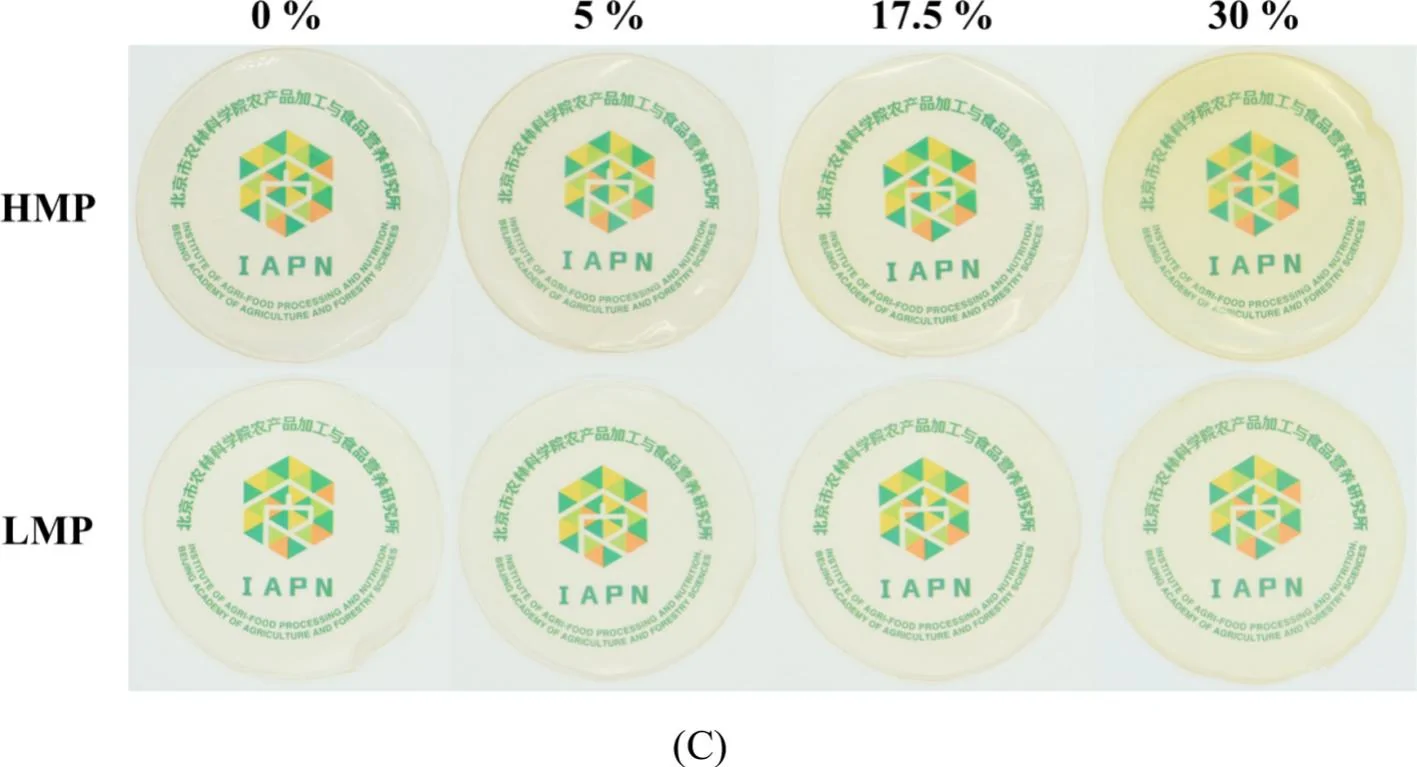
SEM images of surface morphology and cross-sections (A), UV–visible light transmittance spectra (B) and appearance (15 cm in diameter) (C) of pectin-based films activated by *trans*-cinnamaldehyde-loaded nanoemulsion.

The SEM analysis was used to investigate film morphology and distribution of emulsion droplets in the film matrix. As shown in Fig. 2A, the HMP film, irrespective of emulsion incorporation, had smooth surface and compact, homogeneous cross-section, while for the LMP film, the surface was rough and the cross-section was porous and loose. It indicated better compatibility between pectin and emulsion in the HMP film. The incorporation of nanoemulsion induced a modest increase in surface roughness for both HMP and LMP films, causing spherical protrusions on the film surfaces, which is more evident in LMP film. The observed surface heterogeneity may arise from the migration and partial coalescence of nanoemulsion droplets toward the air-film interface when drying (Shi et al., 2016). Similar results have also been reported by Yang et al. (2024) that pectin films containing cellulose nanocrystal-stabilized oregano essential oil emulsion had relatively rough surfaces. However, the films still maintained their structural integrity without cracks, indicating good compatibility between the emulsion and pectin. This could be associated with increased intermolecular cross-linking and non-covalent interactions among pectin, WPI, inulin, and trans-cinnamaldehyde within the film matrix, which is consistent with the spectral shifts observed in FTIR analysis.

#### 3.2.4 Optical properties

Transparency is a pivotal optical attribute in food packaging, as it governs the visual appeal and perceived freshness of wrapped product, thereby affecting consumer acceptance. As shown in Fig. 2B, in the visible light region (400–800 nm), the film without emulsion had the highest transparency. Incorporation of the nanoemulsion resulted in a reduction in transmittance. This is likely due to interactions between proteins and pectin, which increased the films' roughness and light scattering, thereby reducing the transmittance of the films (Silva et al., 2018). However, Fig. 2C shows that nanoemulsion incorporation had negligible impact on the film transparency, an attribute critical for applications in fresh-cut produce packaging. Similarly, neat pectin films had the greatest UV transmission across the 200–400 nm range, reflecting their limited capacity to block ultraviolet radiation. While the composite films added emulsion exhibited notably reduced transmittance. This is because *trans*-cinnamaldehyde has strong absorption characteristics near 285 nm, which greatly reduces the transmittance of UV light (Fan et al., 2023).

Regardless of nanoemulsion incorporation, HMP films consistently exhibited superior optical performance relative to LMP counterparts, characterized by higher visible-light transmittance and significantly enhanced UV-blocking capacity. The former film can effectively block nearly all radiation in the UVC (200–280 nm) and UVB (280–315 nm) ranges and attenuate the majority of UVA (315–400 nm) radiation. This enhanced functionality is because of the denser, more homogeneous microstructure of HMP films as showed in Fig. 2A. Moreover, UV absorbance increased incrementally with nanoemulsion loading, likely attributed to the combined effects of intrinsic chromophoric absorption by *trans*-cinnamaldehyde and light scattering by uniformly dispersed essential oil droplets, both of which contribute to reduced UV transmittance through the films (Ji et al., 2021, Qiu et al., 2025). Collectively, HMP-based films, particularly functionalized with *trans*-cinnamaldehyde-loaded nanoemulsions, delivers an optimal balance of visible transparency and broad band UV protection, offering sufficient transparency for packaging applications while prolonging shelf life of light-sensitive fresh produce.

#### 3.2.5 Film thickness

Thickness is a critical parameter influencing the mechanical and water barrier properties of films. As shown in Table 1, the thickness of pure HMP and LMP films was 83.67 μm and 89.23 μm, respectively. Incorporation of nanoemulsions did not significantly affect the thickness of HMP films overall. However, the HMP film containing 30% nanoemulsion exhibited a significantly lower thickness than those with 5% and 17.5% loadings. This reduction has been previously attributed to a decrease in solids concentration caused by oil phase loss during film formation (Almasi et al., 2020). In contrast, for LMP films, the addition of 5% emulsion showed no effect on thickness, while loadings of 17.5% and 30% resulted in significantly higher thickness compared to the pure LMP film. This increase may be due to the elevated dry matter content introduced by the protein-based nanoemulsion. At equivalent nanoemulsion loadings, LMP-based films consistently displayed greater thickness than their HMP counterparts, suggesting that HMP films possessed a more compact and dense structure.

**Table 1:** Film thickness, permeability of oxygen, carbon dioxide, and water vapor of the pectin-based films activated by *trans*-cinnamaldehyde-loaded nanoemulsion.

Samples	Thickness (μm)	O_2_ (10^−12^ cm^3^·cm·cm^−2^·s^−1^·cmHg^−1^)	CO_2_ (10^−13^ cm^3^·cm·cm^−2^·s^−1^·cmHg^−1^)	water vapor (10^−10^ g·m^−1^·s^−1^·Pa^−1^)
HMP	83.67 ± 6.13^de^	0.92 ± 0.11^b^	1.15 ± 0.13^de^	3.45 ± 0.06^a^
HMP5	90.10 ± 2.29^cd^	0.81 ± 0.20^b^	0.97 ± 0.01^f^	3.31 ± 0.19^ab^
HMP17.5	89.20 ± 2.66^cd^	0.90 ± 0.07^b^	1.97 ± 0.18^bc^	2.89 ± 0.10^cd^
HMP30	81.37 ± 3.95^e^	1.21 ± 0.05^ab^	2.00 ± 0.08^abc^	2.80 ± 0.14^e^
LMP	89.23 ± 3.44^cd^	1.05 ± 0.05^ab^	1.67 ± 0.17^cd^	3.10 ± 0.16^bc^
LMP5	93.77 ± 5.01^bc^	1.17 ± 0.24^ab^	2.25 ± 0.51^ab^	2.97 ± 0.03^cd^
LMP17.5	97.20 ± 1.11^ab^	1.36 ± 0.26^a^	2.58 ± 0.22^a^	2.78 ± 0.08^e^
LMP30	101.57 ± 2.54^a^	1.16 ± 0.22^ab^	2.28 ± 0.22^ab^	2.88 ± 0.10^cd^

Data are expressed as mean ± standard deviations (*n* = 3). Different lowercase letters in the same column denote significant differences among samples (*p* < 0.05).

#### 3.2.6 Gas permeability

As shown in Table 1, O_2_ permeability of pure HMP and LMP films was 0.92 × 10^−12^ and 1.05 × 10^−12^ cm^3^·cm·cm^−2^·s^−1^·cmHg^−1^, respectively. The emulsion incorporation had no significant effect on the O_2_ permeability. No statistically significant differences in O_2_ permeability were noted across HMP- and LMP-based films at equivalent addition amounts, except that the LMP17.5 sample exhibited a markedly higher value (*p* < 0.05). The CO_2_ permeability of pure HMP and LMP films was 1.15 × 10^−13^ and 1.67 × 10^−13^ cm^3^·cm·cm^−2^·s^−1^·cmHg^−1^, respectively. With increasing nanoemulsion loading, HMP-based films exhibited a first decreasing and then increasing trend in CO_2_ permeability, presumably due to the fact that at low contents, the well-dispersed *trans*-cinnamaldehyde-loaded droplets occupy interstitial spaces within the pectin matrix, promoting a more compact and tortuous network that impedes gas diffusion. However, at higher loadings, droplet aggregation or localized phase separation may introduce microstructural heterogeneity, reducing packing density and creating additional pathways that facilitate gas transport (Jakubska et al., 2025). Conversely, LMP films displayed a progressive increase in CO_2_ permeability with nanoemulsion addition. Across equivalent loadings, LMP-based films consistently exhibited higher CO_2_ permeability than HMP counterparts (*p* < 0.05), which could be due to the differences in network architecture. Specifically, the electrostatic repulsion between the negatively charged nanoemulsion droplets (as supported by the ζ-potential data in Section 3.1) and the highly carboxylated LMP chains hindered the formation of a dense network, yielding a more porous and heterogeneous microstructure. In contrast, the stronger hydrophobic associations and hydrogen bonding in HMP promoted a denser, more cohesive matrix, thereby imposing greater diffusional resistance to gas molecules.

Water vapor permeability (WVP) is a critical parameter for evaluating the application of films in food packaging that governed by hydrophobicity and hydrophilicity of film components and the structural compactness of the matrix (Othman et al., 2021). The WVP of pure HMP and LMP was 3.45 and 3.10 (×10^−10^ g·m^−1^·s^−1^·Pa^−1^), respectively. The addition of emulsion markedly reduced WVP of pectin-based films, a finding corroborated by previous study (Almasi et al., 2020). This reduction is likely attributable to the increased hydrophobic residues in the film as evidenced by FTIR analysis. Moreover, it has been reported that the uniform dispersion of nanoemulsion droplets within the film matrix, coupled with increased tortuosity of the diffusion pathways, can extend the effective path length for water vapor transport, thereby reducing permeability (Jomlapeeratikul et al., 2017). The WVP of all films varied between 2.78 and 3.45 (×10^−10^ g·m^−1^·s^−1^·Pa^−1^). This range is comparable to that reported for *Dictyophora rubrovalvata* polysaccharide-incorporated pectin films (2.69–3.61 × 10^−10^ g·m^−1^·s^−1^·Pa^−1^), and lower compared to montmorillonite/pectin films (19.2–21.9 g·m^−1^·s^−1^·Pa^−1^) and chitosan-emulsion-pectin films (24.1–104.7 g·m^−1^·s^−1^·Pa^−1^), exhibiting that the nanoemulsion-loaded pectin film in this study had superior water barrier properties (Song et al., 2026; Zhang et al., 2023).

#### 3.2.7 Water contact angle (WCA)

**Fig. 3:**
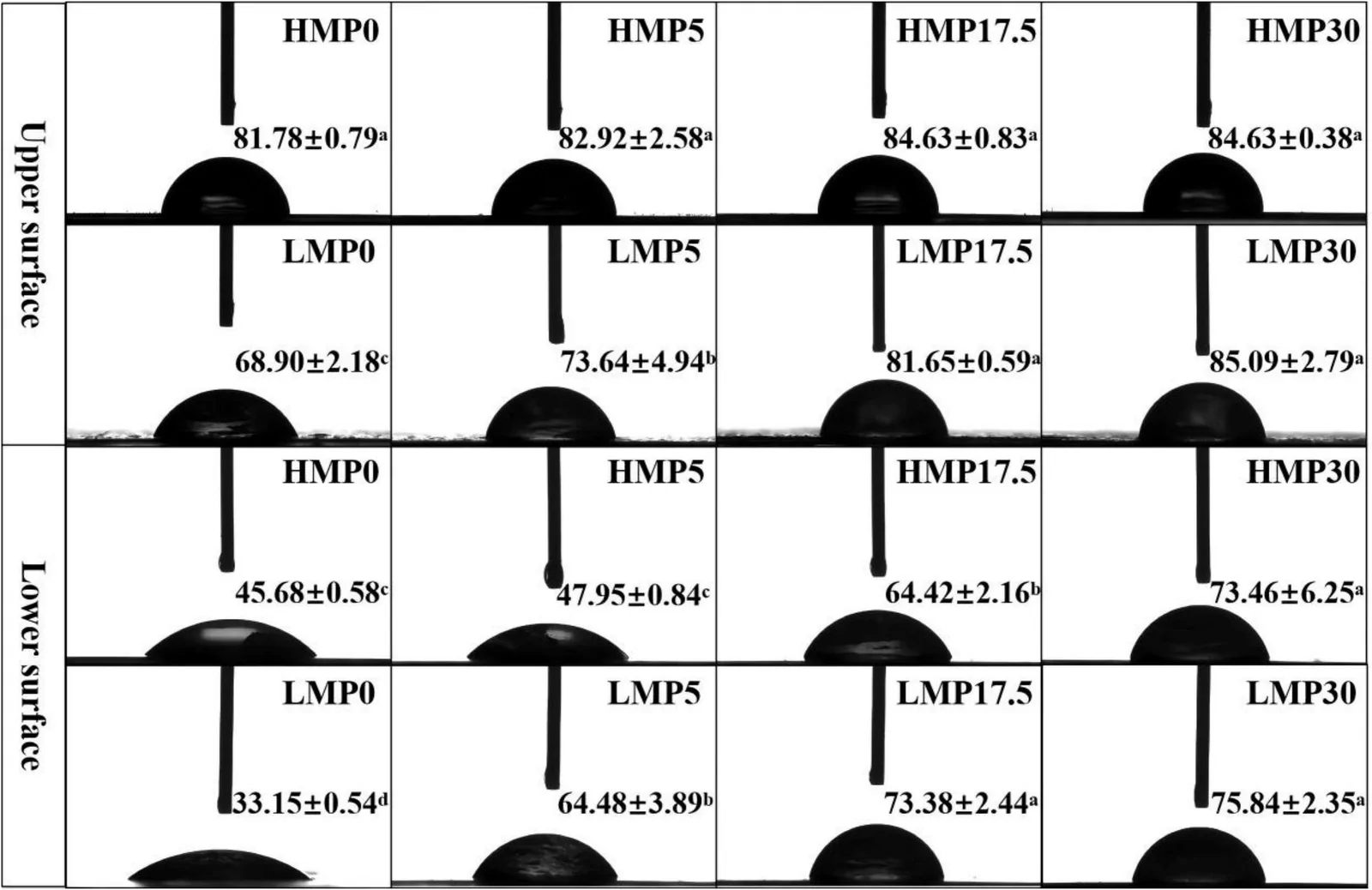
Water contact angles of pectin-based films activated by *trans*-cinnamaldehyde-loaded nanoemulsion. The upper surface was the side exposed to air during drying and the lower surface was the side in contact with the glass Petri dish.

WCA is an important parameter for assessing water resistance and wettability of the biopolymer-based film, with higher values than 65° indicating a “hydrophobic surface” (Zhang et al., 2022). As shown in Fig. 3, the WCA values of upper and lower surfaces of HMP films were 81.78° and 45.68°, respectively, while those of LMP films were 68.9° and 33.15°, respectively. This indicates that the surface hydrophobicity of pure HMP film was stronger than that of LMP, which may be due to the higher esterification degree of HMP, resulting in lower hydrophilicity. For all film formulations, the water contact angle of the upper surface was consistently higher than that of the lower surface, a phenomenon also reported in a prior study (Yu et al., 2024). As the incorporation of nanoemulsion, the hydrophobicity of pectin-based films notably improved, which was related to the increase in hydrophobic amino acids as the emulsion was added (Kasaai, 2018).

#### 3.2.8 Release of *trans*-cinnamaldehyde

**Fig. 4:**
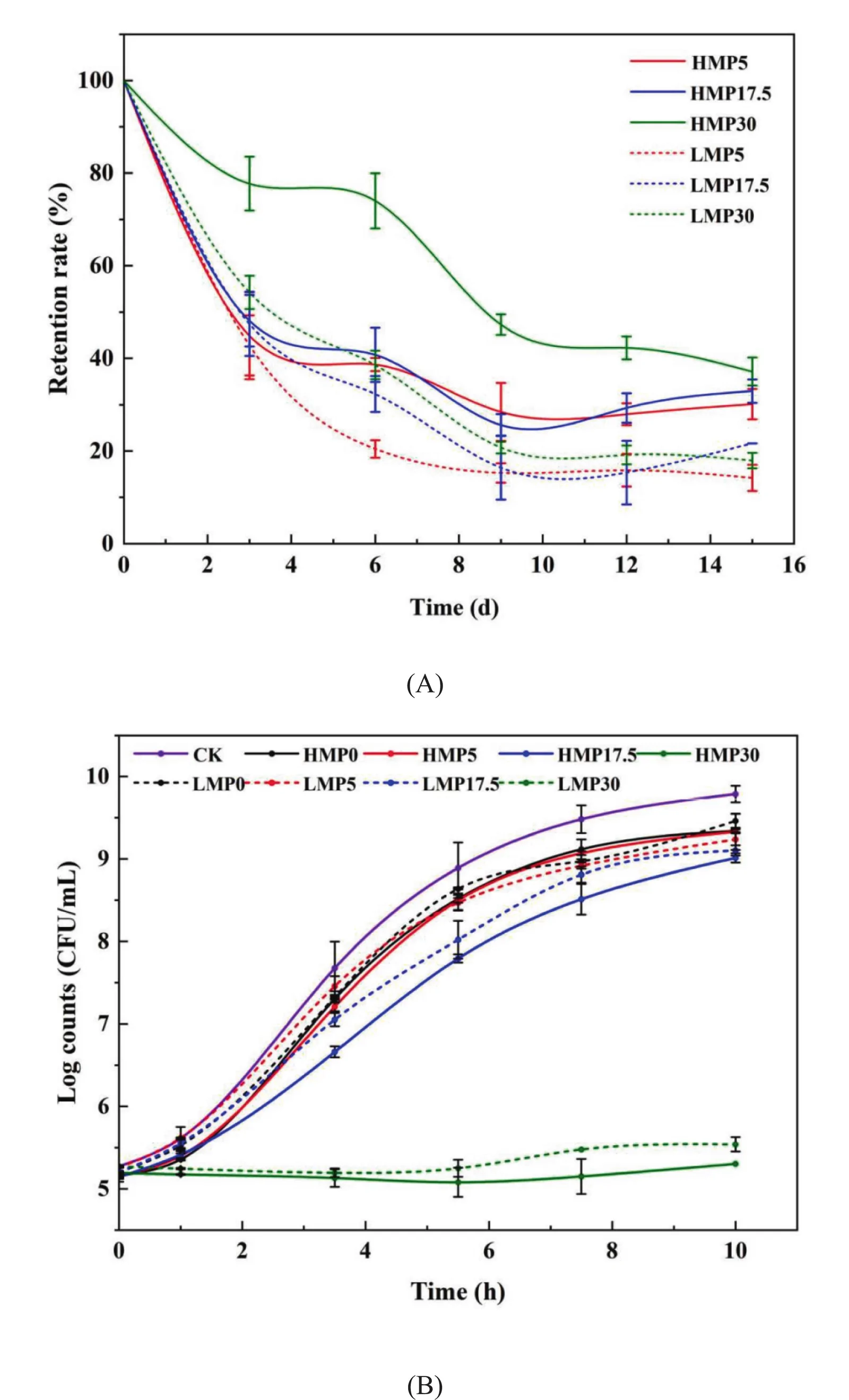
Release of *tran*s-cinnamaldehyde (A) and antimicrobial activity (B) of the pectin-based films activated by *trans*-cinnamaldehyde-loaded nanoemulsion.

The retention of trans-cinnamaldehyde within the films decreased over time, indicating that trans-cinnamaldehyde in the films gradually migrated to the headspace (Fig. 4A). The sustained-release effect became more pronounced with increasing emulsion concentration. The 30% emulsion ratio appeared to provide the greatest protection for the antimicrobial agent, contributing to longer duration of the films' antibacterial properties. The sustained release of *trans*-cinnamaldehyde may be attributed to enhanced interaction between nanoemulsion and pectin molecules in the film matrix, as the FTIR and XRD results showed (Zhu et al., 2025). Compared with LMP, HMP film had higher retention rate of *trans*-cinnamaldehyde at the same concentration, which could be due to the higher proportion of hydrophobic groups (methylated carboxyl group from pectin and hydrophobic amino acids from WPI) which strengthen hydrophobic interactions with the aromatic aldehyde moiety of *trans*-cinnamaldehyde, and the denser network structure of HMP, reinforced by extensive electrostatic interactions and hydrogen bonding between WPI and pectin, may further restrict molecular mobility and reduce volatile loss during storage. Moreover, the HMP film had more compact and homogeneous texture (as indicated by SEM results), which likely contributed to slower release kinetics of the encapsulated essential oil (Muhoza et al., 2019). These results indicate that the presence of the nanoemulsion in the film matrix can slow down the migration of *trans*-cinnamaldehyde to the external environment, and the HMP-based film had better sustained releasing capacity, thereby enabling effective suppression of microbial proliferation and contributing to extended shelf life of fresh-cut chili peppers.

#### 3.2.9 Antibacterial activity

*Enterobacter cloacae,* a prevalent member of the Enterobacteriaceae family, has been identified as a key spoilage bacterium in a range of fresh and minimally processed vegetables, including garlic (Li et al., 2022), potato (El-Hefny et al., 2018), and fresh-cut chili peppers (Li et al., 2025b), where it accelerates textural softening and quality deterioration. Fig. 4B shows the inhibitory effect of pectin-based films on *E. cloacae*. Pure pectin film exhibited minimal antibacterial effect, whereas incorporation of the *trans*-cinnamaldehyde-loaded nanoemulsion can enhance the antimicrobial activity of the resulting composite films. The antibacterial performance increased in a dose-dependent manner with nanoemulsion loading, with the 30% emulsion formulation showing the most effective inhibition of *Enterobacter cloacae* growth. Notably, at equivalent nanoemulsion concentrations, HMP-based films had stronger antibacterial ability at the same emulsion level, which was related to its better sustained-release capacity of *trans*-cinnamaldehyde in the films.

### 3.3 Preservation performance on fresh-cut chili peppers

#### 3.3.1 Appearance

**Fig. 5:**
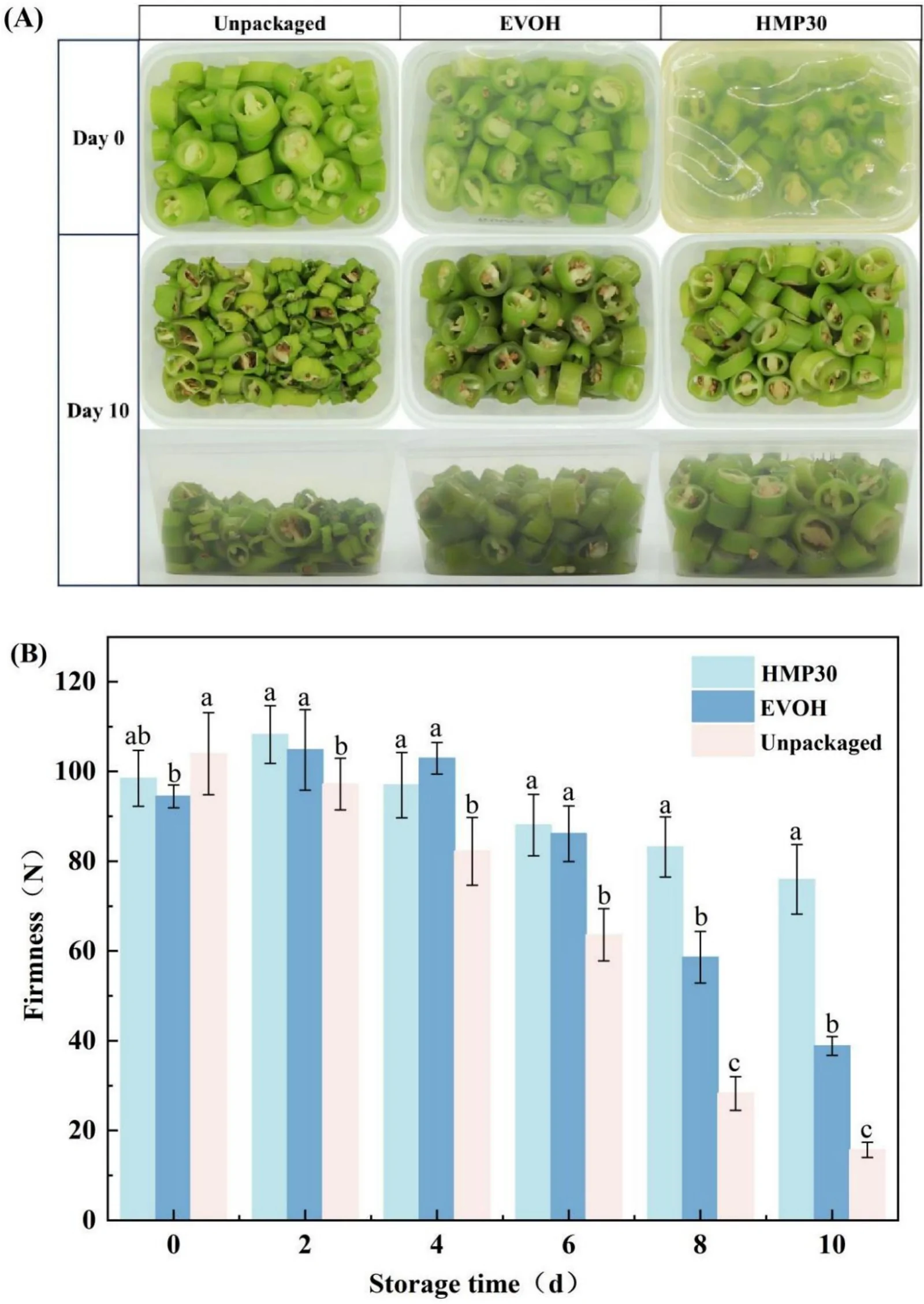
Changes in appearance (A) and firmness (B) of fresh-cut chili pepper stored in different packaging films at 10 °C for 10 days. Different letters within the same storage day indicate significant differences (*p* < 0.05).

Based on the above performance characterization, HMP films with 30% emulsion addition were selected for fresh-cut chili pepper preservation. As shown in Fig. 5A, fresh peppers were bright green in color, with no browning of the seeds and good appearance. After 10 days of storage, the samples unpackaged were severely shriveled, with browning of the seeds. The commercial EVOH-packaged peppers showed water-soaked appearance, browning of the seeds and severe water exudation. In contrast, fresh-cut chili peppers packaged in nanoemulsion incorporated pectin films maintained good overall appearance quality, with no wilting or softening. These results indicate that pectin-based films loaded with *trans*-cinnamaldehyde nanoemulsion have good preservation performance and can effectively delay quality deterioration of fresh-cut chili peppers.

#### 3.3.2 Texture

Firmness is one of the most important quality attributes for fresh-cut chili peppers, serving as a primary indicator of tissue integrity and freshness that significantly influences consumers' acceptance (Li et al., 2025b). The samples without packaging showed the fastest loss of firmness, followed by the EVOH group (Fig. 5B). Compared to these two groups, samples packaged in pectin based-film had significantly higher firmness throughout the whole storage (*p* < 0.05), indicating that pectin film activated nanoemulsion could effectively delay texture loss in fresh-cut chili pepper. This is likely due to its suitable gas permeability and excellent antibacterial properties. Consequently, the composite film has great potential as a biodegradable active packaging material with antibacterial properties.

## 4 Conclusion

In the present work, pectin films activated by nanoemulsion-stabilized *trans*-cinnamaldehyde were successfully prepared. The incorporation of *trans*-cinnamaldehyde emulsions effectively improved the functional properties of pectin-based films, enabling their application as antibacterial active packaging for fresh-cut chili peppers. Increasing the emulsion content significantly enhanced surface hydrophobicity, water vapor and ultraviolet barrier capacities, *trans*-cinnamaldehyde retention, and antibacterial activity. Compared with LMP-based films, HMP films exhibited superior UV shielding, hydrophobicity, sustained release, and antimicrobial performance. Particularly, HMP film containing 30% emulsion was screened and effectively delayed deterioration and preserved the texture attribute of fresh-cut chili peppers. These findings underscore the potential of HMP-based films incorporating *trans*-cinnamaldehyde nanoemulsions as high-performance active packaging solutions for maintaining quality of fresh-cut produce. Although the superior performance of HMP-based films is primarily linked to its higher degree of esterification, it should be acknowledged that commercial pectin sources inherently differ in other structural features, such as molecular weight, neutral sugar composition, and residual protein. These variations may also contribute to the observed differences in film properties, highlighting the need for further investigations using structurally well-defined pectin fractions.

## CRediT authorship contribution statement

**Zudi Li:** Writing – original draft, Visualization, Supervision, Software, Methodology, Investigation, Data curation, Conceptualization. **Xiaoyan Zhao:** Validation, Funding acquisition, Formal analysis. **Qingyu Yang:** Investigation, Formal analysis, Data curation. **Dan Wang:** Data curation. **Pan Wang:** Supervision. **Shuang Zhao:** Investigation. **Wenting Zhao:** Writing – review & editing, Visualization, Funding acquisition, Conceptualization.

## Declaration of competing interest

The authors declare that they have no known competing financial interests or personal relationships that could have appeared to influence the work reported in this paper.

## Data Availability

The authors do not have permission to share data.
