# Supplementary material for: Cinnamaldehyde nanoemulsion-loaded pectin films for delaying quality deterioration of fresh-cut chili pepper during refrigerated storage: Influence of pectin esterification and nanoemulsion concentration
Source: Food Chem X. 2026 Sep 14;39:104469. doi: 10.1016/j.fochx.2026.104469 (PMC13602689; doi:10.1016/j.fochx.2026.104469)
Supplement: Supplementary file 1 [file mmc1.docx]

**Fig. S1.** Particle size distribution of cinnamaldehyde-loaded nanoemulsion.
